# First-Time Parents Are Not Well Enough Prepared for the Safety of Their Infant

**DOI:** 10.1371/journal.pone.0058062

**Published:** 2013-03-06

**Authors:** Mirjam E. J. van Beelen, Tinneke M. J. Beirens, Paul den Hertog, Eduard F. van Beeck, Hein Raat

**Affiliations:** 1 Department of Public Health, Erasmus University Medical Center, Rotterdam, The Netherlands; 2 Dutch Association for Youth Health Care Physicians, Utrecht, The Netherlands; 3 Consumer Safety Institute, Amsterdam, The Netherlands; The University of Queensland, Australia

## Abstract

**Background:**

Unintentional falls and poisonings are major causes of death and disability among infants. Although guidelines are available to prevent these injuries, safety behaviours are not performed by parents, causing unnecessary risks. Little is known about safety behaviours of first-time parents and whether they behave according to these guidelines.

**Aims/Objectives/Purpose:**

The objective of this study was to compare safety behaviours of first-time parents with those of non-first-time parents and to determine correlates of unsafe behaviour of parents of infants. We used self-report questionnaires to assess safety behaviours in a cross-sectional study sample.

**Methods:**

A total of 1439 parents visiting a preventive youth healthcare centre in the Netherlands were invited to complete a questionnaire with regard to the prevention of falls and poisonings. Parents were categorized into first-time parents and non-first-time parents. Correlates of parents' child safety behaviours were determined using multiple logistic regression analyses.

**Results/Outcome:**

Most respondents were mothers (93.2%); 48.2% of families were first-time parents. The mean age of the infants was 7.2 months (SD 1.1; range 4–12), 51.8% were boys, and 34.5% of infants could crawl. First-time parents were more likely not to have a stair gate installed (OR 16.46; 95% CI 12.36–21.93); were more likely to store cleaning products unsafely (OR 4.55; 95% CI 3.59–5.76); and were more likely to store medicines unsafely (OR 2.90; 95% CI 2.31–3.63) than non-first-time parents. First-time parents were more likely to not have a window guard installed (OR 1.52; 95% CI 1.08–2.15) (all *P*<0.05).

**Discussion/Conclusion:**

First-time parents are not well prepared for the safety of their infant, causing unnecessary risks. The various parents' safety behaviours were influenced by different variables, for example, age of the infant, crawling of the infant, mother's educational level, mother's ethnicity, self-efficacy, vulnerability, severity.

## Introduction

Unintentional injuries, such as falls and poisonings, are the fifth leading cause of death among infants [1]. They are also a major source of morbidity and loss of quality of life [2]–[4]. Each year worldwide 1.9 in 100,000 children under 20 years of age die due to falls, and 1.8 in 100,000 children die due to poisonings [2].

The American Academy of Pediatrics (AAP) provides specific informative tools for parents about the safety measures they can take for infants from birth to twelve months of age [5]. To prevent falls, parents are advised to install and always use stair gates on stairs and to install window guards. To prevent poisonings, they are advised to keep household products such as cleaners and chemicals, and medicines out of sight and reach [5].

Parents with several children have often taken various safety measures [6]–[8]. However, when they have their first child many safety measures still need to be taken. It is important for first-time parents to be prepared to raise their infant in a safe environment. Little is known about the safety behaviours of first-time parents en whether they behave according to the recommendations of the AAP. For the purpose of developing strategies to reduce the number of injuries from falls and poisonings, it is important to know which preventive actions first-time parents actually take. It is also useful to know which parent and child characteristics and other determinants are associated with these preventive actions, in order to develop effective intervention strategies. More information is needed on these determinants related to protecting infants against unintentional injuries in the home.

Behaviours are influenced by a complex, interrelated set of determinants, which include various demographic and psychosocial factors. To assess the influence of underlying psychosocial factors on behaviours, the Protection Motivation Theory (PMT) has been proven reliable in predicting behaviours [9]–[11]. Protection Motivation Theory is a framework particularly suited to interventions of protective, precautionary behaviours. According to this theory, safe behaviour is directly influenced by protection motivation, which is the result of an evaluation of environmental and personal factors. It posits that the probability of safe behaviour, in this case preventing falls and poisoning, is increased by four beliefs: 1) the personal abilities and self-confidence to always use a stair gate and store cleaning products and medicines safe, self-efficacy; 2) the perception of the adaptive response to use a stair gate and store cleaning products and medicines safe, response efficacy; 3) the perception of personal relevance regarding falls from the staircase or of ingestion of cleaning products and medicines, vulnerability; and 4) the perception of severity in the event of a fall on the stairs or of ingesting cleaning products or medicines. In this study we used demographic variables as well as PMT constructs to assess the influence of underlying psychosocial factors on parents' child safety behaviours.

The objective of this study was to compare safety behaviours of first-time parents with safety behaviours of non-first-time parents and to determine correlates of unsafe behaviour parents of infants. We used self-report questionnaires on safety behaviour to assess these safety behaviours in a cross-sectional study sample.

## Materials and Methods

### Participants and recruitment

The present study used data obtained at enrolment in the ‘BeSAFE’ study, a randomized controlled trial which aims to assess the effects of internet-based, tailored safety information combined with personal counselling on parents' child safety behaviours, as described in detail elsewhere [12]. An opportunity sample of five preventive youth health care centres in the Netherlands invited a total of 3147 parents of infants aged 5 to 12 months old (one parent per family) to participate in the study between 2009 and 2010. These five youth health care centres were located in urban and rural areas. Written informed consent was provided by 45.7% (n = 1439), who also completed the questionnaire.

The Medical Ethics Committee of the Erasmus Medical Center gave a “declaration of no objection” for this study (MEC-2008-370). The ‘BeSAFE’ study was registered in the Dutch Trial Registration (Current Controlled Trials NTR1836).

### Measurements

Parents received written information about the study, were asked to provide informed consent and were asked to complete the questionnaire on home safety. Up to two reminders were sent. Parents were assured of confidentiality and the results were processed anonymously.

The questionnaire assessed family, infant and housing characteristics, parents' child safety behaviour, and ‘Protection Motivation Theory’-constructs.

#### Family, infant, and housing characteristics

Number of children was assessed and dichotomized as first-time parents (first child in family) and non-first-time parents (second child or more children in family). Parents' educational level was assessed and categorized as ‘high’, ‘intermediate’, and ‘low’; high level being defined as higher professional education or academic higher education; intermediate level as senior secondary vocational education, senior general secondary education or university preparatory education; low educational level being defined as preparatory secondary vocational education or lower [13]. Parents' employment status was assesses and defined as “unemployed” if they had neither a part-time or full-time job. Parents' ethnicity was determined on the basis of their own parents' country of birth (grandparents of the infant). A parent was of Dutch ethnicity if both grandparents had been born in The Netherlands; if one of the grandparents had been born in another Western country, a parent was of other Western ethnicity. If both grandparents had been born in another Western or non-Western country, ethnicity was determined by the grandmother's country of birth [13].

Crawling was assessed and defined as an infant's ability to: “crawl on hands and knees and/or crawl on their tummy and/or shuffle on their bottom”.

Infant's medically attended injury was assessed and categorized as ‘none’ and ‘one or more’; one or more was defined as one or more injuries for which the child was taken to a general practitioner, the emergency department of a hospital, or both.

#### Protection Motivation Theory constructs

The psychosocial correlates of safety behaviour were measured with regard to Protection Motivation Theory constructs [9], [10]. Self-efficacy was measured from −2 = very difficult to +2 = very easy, response efficacy was measured from −2 = not very helpful to +2 = very helpful, vulnerability was measured from −2 = low risk; +2 = high risk, and severity was measured from −2 = not serious; +2 = very serious. All items related to the Protection Motivation Theory constructs were measured on bipolar five-point scales.

#### Parents' child safety behaviour with regard to falls

Parents were asked whether there was a staircase between the floor with the living room and a separate floor with the bedrooms; if so, this was designated as the main staircase. The presence of a stair gate at the top or bottom of the main staircase was assessed. The self-reported frequency of closing the stair gate of the main staircase was measured on a five-point scale (‘never’ to ‘always’); adequate use was defined as ‘always closing the stair gate’. The presence of windows which could be opened, below the height of 1.20 m, was assessed, and parents were asked whether they had window guards on at least one of such windows.

#### Parents' child safety behaviour with regard to poisoning

Parents were asked where they stored their cleaning products and medicines. ‘Unsafe’ storage of cleaning products was defined as stored in the bathroom, kitchen or shed/garage on the floor or in a cupboard without a lock, at a height below 1.50 m. ‘Unsafe’ storage of medicines was defined as stored in the bathroom, kitchen or bedroom on the floor or in a cupboard without a lock, at a height below 1.50 m.

### Statistical analyses

Categorical data were described using percentages and continuous data using mean (SD). Differences in the proportions and means of all potential correlates were tested by chi-square test and Mann-Whitney *U* test. First-time parents' safety behaviours and non-first-time parents' safety behaviours were compared between infants who could not crawl and those who could crawl.

To determine significant correlates of parents' safety behaviour, multiple logistic regression analyses were performed, with unsafe behaviour as the dependent variable and various factors (demographic and Protection Motivation Theory constructs) as independent variables. Five different sets of multiple logistic regression analyses were conducted, first for respondents who indicated the absence of a stair gate on their main stairs, and second for the sub-group of respondents who had a stair gate but did not use it adequately. A third set described the correlates of the absence of a window guard on windows below the height of 1.20 m. A fourth set was conducted with regard to the unsafe storage of cleaning products and a fifth on the unsafe storage of medicines. In model 1 of every set the number of children (e.g. first-time parents vs. non-first-time parents, with regard to unsafe behaviour was entered. In model 2 other demographic variables that were considered to be more distal, these were non-modifiable potential correlates, were entered. Subsequently, in model 3, Protection Motivation Theory constructs were entered into the models.

Statistical analyses were performed using SPSS 17.0 (SPSS Inc., Chicago, IL.).

## Results

### Family, infant, and housing characteristics

Most respondents were mothers (93.2%); 48.2% were first-time parents; 97.2% of the families included two parents. Fewer first-time mothers were unemployed than non-first-time mothers (13.4% vs. 23.2% respectively; *P*<0.001) (Table 1). The mean age of the infants was 7.2 months (SD 1.1; range 4–12 months); 51.8% were boys; 34.5% could crawl, and 0.5% could walk independently. A main staircase was present in 86.6% of houses; 36.6% of houses had a window below a height of 1.20 m, which could be opened. Fewer first-time parents (82.7%) had a main staircase present than non-first-time parents (90.2%) (*P*<0.001).

**Table 1:** Family, infant and housing characteristics, divided by number of children (n = 1439).

		Total (%) (Unless otherwise specified)	First-time parents (%) (Unless otherwise specified)	Non-first-time parents^4^ (%) (Unless otherwise specified)
		n = 1439	n = 693	n = 746
**Family characteristics**				
Mother was respondent		93.2	92.4	94.0
Mother's educational level	High^1^	39.0	39.3	38.7
	Intermediate^2^	44.2	45.2	43.2
	Low^3^	16.8	15.5	18.1
Father's educational level	High^1^	36.1	39.3	37.3
	Intermediate^2^	40.9	45.2	38.7
	Low^3^	23.0	15.5	24.0
Mother's employment status	Unemployed	18.5	13.4	23.2***
Father's employment status	Unemployed	4.4	3.5	5.3
Mother's ethnicity	Dutch	86.7	87.0	86.5
	Other Western	4.6	5.5	3.8
	Non-Western	8.7	7.5	9.8
Father's ethnicity	Dutch	86.8	87.0	86.6
	Other Western	4.7	5.7	3.8
	Non-Western	8.5	7.3	9.6
Single parent	Yes	2.8	2.5	3.1
**Infant characteristics**				
Infant's age in months	Mean (SD); range	7.2 (1.1); 4–12	7.2 (1.0); 4–12	7.2 (1.1); 4–12
Gender	Boy	51.8	55.3	48.5*
Infant could crawl	Yes	34.5	34.8	34.2
Infant could walk independently	Yes	0.5	0.4	0.5
Lifetime prevalence of medically attended unintentional injury	One or more	2.8	2.2	3.4
**Housing characteristics**				
Presence of main staircase in the house	Yes	86.6	82.7	90.2***
Presence of windows below theheight of 1.20 m (which can be opened)	Yes	36.6	36.4	36.8

1 High educational level: at least higher professional education.

2 Intermediate educational level: senior secondary vocational education, senior general secondary education and university preparatory education.

3 Low educational level: preparatory secondary vocational education or less.

4 Differences in characteristics of first-time parents and non-first-time parents evaluated by chi-square test or Mann-Whitney U-test:

* Significant at the 0.05 level,

** significant at the 0.01 level,

*** significant at the 0.001 level.

### Safety behaviour of first-time parents

If their infant could not crawl, more first-time parents had not installed a stair gate (89.4%) than first-time parents with an infant that could crawl (75.0%) (*P*<0.05) (Table 2). If their infant could not crawl, more first-time parents stored medicines unsafely (54.3%) than first-time parents with an infant that could crawl (43.6%) (*P*<0.05). There were no differences in the safety behaviours between non-first-time parents whose infant could crawl and those whose infants could not crawl (*P*>0.05).

**Table 2:** First-time and non-first-time parents' safety behaviour relevant to the prevention of falls and poisonings, compared between infants who cannot crawl and infants who can crawl (n = 1439).

		First-time parents			Non-first-time parents		
	Total group (%)	Infant cannotCrawl (%)	Infant can crawl (%)	*P*-value±	Infant cannotCrawl (%)	Infant canCrawl (%)	*P*-value±
**Falls**							
Main staircase in the house 1	n = 1245	n = 388	n = 184		n = 453	n = 219	
Absence of stair gate	52.6	**89.4**	**75.0**	**<0.001**	26.0	23.7	0.52
Presence of stair gate	47.4	**10.6**	**25.0**		74.0	76.3	
Stair gate use 1	n = 590	n = 41	n = 46		n = 335	n = 167	
No adequate use	41.1	42.5	34.8	0.46	42.0	40.4	0.72
Adequate use	58.9	57.5	65.2		58.0	59.6	
Windows below 1.20 m 1	n = 526	n = 170	n = 82		n = 183	n = 91	
No window guard	55.3	58.2	65.9	0.25	51.9	47.3	0.47
Window guard	44.7	41.8	34.1		48.1	52.7	
**Poisonings**							
Storage of cleaning products	n = 1439	n = 451	n = 241		n = 490	n = 255	
Unsafe storage	60.3	78.7	75.5	0.16	44.6	43.7	0.96
Safe storage	37.0	18.4	23.2		53.0	52.4	
Unknown storage	2.6	2.9	1.2		2.5	3.9	
Storage of medicines	n = 1439	n = 451	n = 241		n = 490	n = 255	
Unsafe storage	38.2	**54.3**	**43.6**	**0.01**	27.8	24.1	0.33
Safe storage	54.1	**39.7**	**48.1**		64.2	66.4	
Unknown storage	7.7	**6.0**	**8.3**		8.0	9.5	

1 Only when situation is applicable.

± Differences between infants who cannot crawl and can crawl evaluated by Chi-square test.

Note: Bold numbers indicate significant *P*-values.

First-time parents were more likely not to have a stair gate installed (OR 16.46; 95% CI 12.36–21.93); were more likely to store cleaning products unsafely (OR 4.55; 95% CI 3.59–5.76); and were more likely to store medicines unsafely (OR 2.90; 95% CI 2.31–3.63) than non-first-time parents (all *P*<0.05) (Table 3). Furthermore first-time parents were more likely to not have a window guard installed (OR 1.52; 95% CI 1.08–2.15) than non-first-time parents (*P*<0.05) (Tables 3, 4, 5, 6, 7).

**Table 3:** Odds ratios (OR) and 95% confidence intervals from multiple logistic regression analyses with reported absence of stair gate as dependent variable and number of children (Model 1), other demographic variables (Model 2) and Protection Motivation Theory (PMT) variables (Model 3) as independent factors in a subgroup of parents with a main staircase present in their house (n = 1245).

		Absence of stair gate		
		Model 1	Model 2	Model 3
		OR (95% CI)	OR (95% CI)	OR (95% CI)
**Demographic variables**				
Number of children	First-time parents	**16.46 (12.36–21.93)** ***	**17.53 (13.04–23.56)** ***	**19.60 (14.36–26.75)** ***
	Non-first-time parents	1.00	1.00	1.00
Infant's age	0–6 months		0.78 (0.53–1.15)	0.79 (0.53–1.18)
	6–12 months		1.00	1.00
Infant's gender	Girl		1.07 (0.80–1.41)	1.11 (0.83–1.48)
	Boy		1.00	1.00
Infant can crawl	No		**1.66 (1.22–2.27)** **	**1.69 (1.23–2.32)** **
	Yes		1.00	1.00
Mother's educational level	High		1.24 (0.81–1.91)	1.13 (0.72–1.76)
	Intermediate		1.17 (0.76–1.79)	1.14 (0.73–1.77)
	Low		1.00	1.00
Mother's ethnicity	Non-Western		**1.87 (1.02–3.43)** *	1.56 (0.85–2.87)
	Other Western		1.65 (0.81–3.33)	1.58 (0.77–3.24)
	Dutch		1.00	1.00
**PMT constructs**				
Self-efficacy	−2, +2			n.a.
Response efficacy	−2, +2			n.a.
Vulnerability	−2, +2			**1.56 (1.35–1.80)** ***
Severity	−2, +2			**0.76 (0.61–0.94)** *
Nagelkerke R^2^		0.42	0.44	0.47

n.a. not assessed.

* Significant at the 0.05 level,

** significant at the 0.01 level,

*** significant at the 0.001 level.

Note: Bold numbers indicate significant *P*-values.

**Table 4:** Odds ratios (OR) and 95% confidence intervals from multiple logistic regression analyses with no adequate use of the stair gate as dependent variable and number of children (Model 1), other demographic variables (Model 2) and Protection Motivation Theory (PMT) variables (Model 3) as independent factors in a subgroup of parents with a stair gate present at their staircase (n = 590).

		No adequate use of stair gate		
		Model 1	Model 2	Model 3
		OR (95% CI)	OR (95% CI)	OR (95% CI)
**Demographic variables**				
Number of children	First-time parents	0.87 (0.55–1.40)	0.89 (0.55–1.46)	0.83 (0.47–1.47)
	Non-first-time parents	1.00	1.00	1.00
Infant's age	0–6 months		0.81 (0.49–1.33)	0.84 (0.48–1.48)
	6–12 months		1.00	1.00
Infant's gender	Girl		0.89 (0.63–1.25)	0.70 (0.48–1.03)
	Boy		1.00	1.00
Infant can crawl	No		1.05 (0.73–1.51)	0.97 (0.65–1.46)
	Yes		1.00	1.00
Mother's educational level	High		**2.77 (1.61–4.78)** ***	**2.99 (1.58–5.65)** ***
	Intermediate		**2.24 (1.31–3.84)** **	**2.32 (1.24–4.35)** **
	Low		1.00	1.00
Mother's ethnicity	Non-Western		0.94 (0.43–2.05)	0.69 (0.29–1.61)
	Other Western		**2.91 (1.12–7.51)** *	2.98 (0.99–9.02)
	Dutch		1.00	1.00
**PMT constructs**				
Self-efficacy	−2, +2			**0.28 (0.20–0.38)** ***
Response efficacy	−2, +2			0.79 (0.53–1.17)
Vulnerability	−2, +2			1.13 (0.92–1.38)
Severity	−2, +2			0.98 (0.72–1.32)
Nagelkerke R^2^		0.001	0.05	0.28

* Significant at the 0.05 level,

** significant at the 0.01 level,

*** significant at the 0.001 level.

Note: Bold numbers indicate significant *P*-values.

**Table 5:** Odds ratios (OR), 95% confidence intervals and explained variance (Nagelkerke R^2^) from multiple logistic regression analyses with reported absence of window guard as dependent variable and number of children (Model 1), other demographic variables (Model 2) and Protection Motivation Theory (PMT) variables (Model 3) as independent factors in a subgroup of parents with windows that could be opened in their house (n = 526).

		Absence of window guard		
		Model 1	Model 2	Model 3
		OR (95% CI)	OR (95% CI)	OR (95% CI)
**Demographic variables**				
Number of children	First-time parents	**1.52 (1.08–2.15)** *	**1.60 (1.13–2.27)** **	**1.60 (1.13–2.28)** **
	Non-first-time parents	1.00	1.00	1.00
Infant's age	0–6 months		0.83 (0.51–1.35)	0.83 (0.51–1.35)
	6–12 months		1.00	1.00
Infant's gender	Girl		1.21 (0.85–1.71)	1.21 (0.85–1.72)
	Boy		1.00	1.00
Infant can crawl	No		0.89 (0.60–1.31)	0.89 (0.60–1.31)
	Yes		1.00	1.00
Mother's educational level	High		1.69 (1.00–2.85)	1.69 (1.00–2.86)
	Intermediate		1.56 (0.93–2.62)	1.56 (0.93–2.62)
	Low		1.00	1.00
Mother's ethnicity	Non-Western		0.99 (0.54–1.83)	0.99 (0.54–1.83)
	Other Western		0.74 (0.29–1.88)	0.74 (0.29–1.88)
	Dutch		1.00	1.00
**PMT constructs**				
Self-efficacy	−2, +2			1.00 (1.00-1.00)
Response efficacy				n.a.
Vulnerability				n.a.
Severity				n.a.
Nagelkerke R^2^		0.01	0.03	0.03

n.a. not assessed.

* Significant at the 0.05 level,

** significant at the 0.01 level,

*** significant at the 0.001 level.

Note: Bold numbers indicate significant *P*-values.

**Table 6:** Odds ratios (OR), 95% confidence intervals and explained variance (Nagelkerke R^2^) from multiple logistic regression analyses with reported unsafe storage of cleaning products as dependent variable and number of children (Model 1), other demographic variables (Model 2) and Protection Motivation Theory (PMT) variables (Model 3) as independent factors (n = 1439).

		Unsafe storage of cleaning products		
		Model 1	Model 2	Model 3
		OR 95% CI)	OR (95% CI)	OR 95% CI)
**Demographic variables**				
Number of children	First-time parents	**4.55 (3.59–5.76)** ***	**4.69 (3.68–5.98)** ***	**4.53 (3.53–5.82)** ***
	Non-first-time parents	1.00	1.00	1.00
Infant's age	0–6 months		1.05 (0.76–1.47	1.09 (0.78–1.53)
	6–12 months		1.00	1.00
Infant's gender	Girl		0.99 (0.79–1.26)	0.96 (0.76–1.23)
	Boy		1.00	1.00
Infant can crawl	No		1.15 (0.89–1.48)	1.09 (0.83–1.41)
	Yes		1.00	1.00
Mother's educational level	High		**2.22 (1.58–3.13)** ***	**2.05 (1.43–2.94)** ***
	Intermediate		**1.82 (1.31–2.54)** ***	**1.70 (1.20–2.39)** **
	Low		1.00	1.00
Mother's ethnicity	Non-Western		**1.97 (1.26–3.07)** **	**2.04 (1.29–3.25)** **
	Other Western		**1.89 (1.03–3.49)** *	1.81 (0.97–3.37)
	Dutch		1.00	1.00
**PMT constructs**				
Self-efficacy	−2, +2			**0.74 (0.62–0.88)** ***
Response efficacy	−2, +2			1.03 (0.84–1.27)
Vulnerability	−2, +2			**1.14 (1.01–1.29)** *
Severity	−2, +2			0.98 (0.82–1.17)
Nagelkerke R^2^		0.16	0.19	0.21

* Significant at the 0.05 level,

** significant at the 0.01 level,

*** significant at the 0.001 level.

Note: Bold numbers indicate significant *P*-values.

**Table 7:** Odds ratios (OR), 95% confidence intervals and explained variance (Nagelkerke R^2^) from multiple logistic regression analyses with reported unsafe storage medicines as dependent variable and number of children (Model 1), other demographic variables (Model 2) and Protection Motivation Theory (PMT) variables (Model 3) as independent factors (n = 1439).

		Unsafe storage of medicines		
		Model 1	Model 2	Model 3
		OR (95% CI)	OR (95% CI)	OR (95% CI)
**Demographic variables**				
Number of children	First-time parents	**2.90 (2.31–3.63)** ***	**2.96 (2.35–3.73)** ***	**2.83 (2.23–3.60)** ***
	Non-first-time parents	1.00	1.00	1.00
Infant's age	0–6 months		**0.67 (0.48–0.93)** *	**0.69 (0.50–0.97)** *
	6–12 months		1.00	1.00
Infant's gender	Girl		1.07 (0.85–1.34)	1.09 (0.85–1.38)
	Boy		1.00	1.00
Infant can crawl	No		1.23 (0.96–1.59)	1.16 (0.89–1.50)
	Yes		1.00	1.00
Mother's educational level	High		**2.26 (1.59–3.23)** ***	**1.96 (1.35–2.84)** ***
	Intermediate		**1.53 (1.08–2.17)** *	1.40 (0.98–2.02)
	Low		1.00	1.00
Mother's ethnicity	Non-Western		0.92 (0.59–1.44)	0.90 (0.58–1.41)
	Other Western		1.26 (0.74–2.17)	1.41 (0.80–2.48)
	Dutch		1.00	1.00
**PMT constructs**				
Self-efficacy	−2, +2			**0.72 (0.61–0.85)** ***
Response efficacy	−2, +2			1.15 (0.93–1.42)
Vulnerability	−2, +2			1.15 (1.02–1.29)
Severity	−2, +2			0.95 (0.80–1.14)
Nagelkerke R^2^		0.09	0.12	0.14

* Significant at the 0.05 level,

** significant at the 0.01 level,

*** significant at the 0.001 level.

Note: Bold numbers indicate significant *P*-values.

### Multiple correlates of safety behaviour

Number of children, crawling of their infant, vulnerability, and severity were significantly associated with the absence of a stair gate (*P*<0.05) (Table 3).

Educational level, mother's ethnicity, and self-efficacy were significantly associated with the inadequate use of a stair gate (*P*<0.05) (Table 4). In this model number of children was not associated with the behaviour.

Only number of children was a significant variable (*P*<0.05) of the variance in the absence of a window guard (Table 5).

Number of children, mother's educational level, mother's ethnicity, self-efficacy, and vulnerability were significant variables (*P*<0.05) of the variance in the unsafe storage of cleaning products (Table 6).

Number of children, infant's age, mother's educational level, and self-efficacy were significant variables (*P*<0.05) of the variance in unsafe storage of medicines (Table 7).

## Discussion

The results of this study show that there is a difference in safety behaviour between first-time parents and non-first-time parents. Regarding having a stair gate at the main staircase at the house and storage of medicines, more first-time parents with infants who cannot crawl behave unsafe than first-time parents with an infant that can crawl. Furthermore the results show that different demographic variables are associated with unsafe behaviour of parents of infants. From our study it can be concluded that PMT constructs are applicable to predict the absence of a stair gate, inadequate use of a stair gate, and unsafe storage of cleaning products and medicines.

This study shows that first-time parents don't behave as recommended in the prevention of falls and poisonings. When infants of first-time parents start crawling parents are probably more aware of the dangers in their home, and therefore start showing the required safety behaviour only then when their child is already at risk. Earlier studies show that these parents still do not take enough safety measures to prevent injuries [7], [8]. When infants are able to move around the house, they are able to explore their world. It is recommended to prepare for a safe home environment before infants can move themselves around [5]. Since one cannot predict exactly when each child develops these abilities it is important to start showing these safety behaviours at an early stage.

Especially first-time parents are not well prepared for their crawling infant compared to non-first-time parents. When older siblings are present in the home, safety behaviours with regard to the presence of stair gates are shown more often possibly based on their experience with their older child or children. However parents still do not use the stair gate adequately. Or maybe they stop using the stair gate adequately, because their older sibling can walk the stairs himself.

Earlier study on parents' safety behaviours of Brice, et al showed no significant differences on infant-safe homes between first-time mothers and non-first-time mothers. This study however did not focus on prevention of falls or poisonings [14].

The associations included in our study were similar to the results in previous studies on safety behaviour [6], [15]–[17]. However, to our knowledge, this is the first study to look specifically at first-time parents. First-time parents, infants that could not crawl, parents who perceived lower vulnerability of their child and parents who perceived lower severity were more likely not to have a stair gate present. Besides, when a stair gate is present mothers with an intermediate or high educational level, or mothers with lower self-efficacy are more likely to use the stair gate inadequately. It could be beneficial to aim specific interventions at these parents in order to reduce the number of injures due to falls from stairs.

Injuries from falls from a window especially occur in children aged 0–4 years old, with a peak at one year [18]. In our study we saw that number of children was correlated with absence of a window guard and no other demographic characteristics or PMT constructs. It is therefore important to point prevention strategies at all parents, not families with specific characteristics. It is however recommended to specifically inform first-time parents about the prevention of falls from windows and the use of window guards in order to improve the use of these window guards.

First-time parents, mothers with intermediate or high educational level, Non-Western ethnicity of the mother, lower self-efficacy and higher perceived vulnerability are correlated with unsafe storage of cleaning products. Furthermore first-time parents, younger children, high educational level of the mother and lower self-efficacy correlated with unsafe storage of medicines. These results indicate that the characteristics of parents who behave unsafely vary within the prevention of different types of injuries, in this case poisoning. Despite the decrease in the past years in the number of poisoning related injuries due to improved poisoning prevention strategies, still too many of these injuries occur [19]. Previous studies showed that parents do not adopt the recommendations for safe storage of poisonings [20].

### Methodological considerations

Some limitations of this study need to be addressed. First, because our study relied on self-report of safe and unsafe behaviour by parents, misclassification might have occurred; parents might have given socially desirable answers (overstating their safe behaviour) [21], [22]. This might result in an underestimation of unsafe households, and bias in the assessment of significant correlates.

Of the 1439 participants that completed the questionnaire, only 7% were not born in the Netherlands. We assume that these participants had adequate knowledge of the Dutch language to enable participation in the study and complete the questionnaire. We recommend future studies to address language skills of participants in the study.

Not all PMT constructs were measured on all behaviours, for example self-efficacy and response efficacy in stair gate presence in the house and response efficacy, vulnerability and severity in use of a window guard on windows.

Participation rate in this study, 46%, was low. This study was part of a randomized controlled trial which aims to assess the effects of internet-based, tailored safety information combined with personal counselling. Therefore, participants were invited to complete more than one questionnaire. Also, this study required participants to have access to the Internet. This may have contributed to the low participation rate.

There is no data available on the characteristics of parents who did not wish to participate in this study. It is difficult to ascertain whether the associations found would be different in non-responders.

This study used an opportunity sample of five preventive youth health care centres in the Netherlands. The participation rate and the use of an opportunity sample may have caused limited generalizability of our study results.

### Future research

We suggest to measure safety behaviour and PMT constructs longitudinal in order to investigate when parents change their behaviour and which variables are associated with the change in safety behaviour. Furthermore the study could be extended with home safety observations in order to eliminate possible misclassification.

### Conclusion

First-time parents are not well prepared for the safety of their infant, causing unnecessary unsafe situations. Especially when their infant cannot crawl yet, parents behave unsafely on not having a stair gate and the storage of medicines. The various parents' safety behaviours were influenced by different variables, e.g. age of the infant, crawling of the infant, mother's educational level, mother's ethnicity, self-efficacy vulnerability, and severity. These variables could be taken into account when providing safety information to parents.
